# Accurate and fast implementation of soybean pod counting and localization from high-resolution image

**DOI:** 10.3389/fpls.2024.1320109

**Published:** 2024-02-20

**Authors:** Zhenghong Yu, Yangxu Wang, Jianxiong Ye, Shengjie Liufu, Dunlu Lu, Xiuli Zhu, Zhongming Yang, Qingji Tan

**Affiliations:** ^1^ College of Robotics, Guangdong Polytechnic of Science and Technology, Zhuhai, China; ^2^ Department of Network Technology, Guangzhou Institute of Software Engineering, Conghua, China; ^3^ School of Electronics and Information Engineering, Wuyi University, Jiangmen, China; ^4^ College of Business, Guangzhou College of Technology and Business, Foshan, China; ^5^ School of Mechanical Engineering, Guangxi University, Nanning, China

**Keywords:** soybean pod, convolutional network, computer vision, counting and locating, dense objects

## Abstract

**Introduction:**

Soybean pod count is one of the crucial indicators of soybean yield. Nevertheless, due to the challenges associated with counting pods, such as crowded and uneven pod distribution, existing pod counting models prioritize accuracy over efficiency, which does not meet the requirements for lightweight and real-time tasks.

**Methods:**

To address this goal, we have designed a deep convolutional network called PodNet. It employs a lightweight encoder and an efficient decoder that effectively decodes both shallow and deep information, alleviating the indirect interactions caused by information loss and degradation between non-adjacent levels.

**Results:**

We utilized a high-resolution dataset of soybean pods from field harvesting to evaluate the model’s generalization ability. Through experimental comparisons between manual counting and model yield estimation, we confirmed the effectiveness of the PodNet model. The experimental results indicate that PodNet achieves an R^2^ of 0.95 for the prediction of soybean pod quantities compared to ground truth, with only 2.48M parameters, which is an order of magnitude lower than the current SOTA model YOLO POD, and the FPS is much higher than YOLO POD.

**Discussion:**

Compared to advanced computer vision methods, PodNet significantly enhances efficiency with almost no sacrifice in accuracy. Its lightweight architecture and high FPS make it suitable for real-time applications, providing a new solution for counting and locating dense objects.

## 1 Introduction

Soybeans are renowned for their high protein content and have become a staple in global nutrition. Approximately 40% of the weight of soybean seeds consists of protein, making soybeans a primary source of protein for vegetarians and strict vegans (Wiles and Schweizer, 1999). Pod count is a critical factor influencing soybean yield. In traditional agriculture, pod counting is typically done manually (Uzal et al., 2018). Manual counting, however, is subjective, tedious, error-prone, and inefficient due to human fatigue. Over the past few decades, agricultural practitioners have been attempting to automate this task using machine learning methods (Ye et al., 2013; Yu et al., 2013). Unfortunately, these methods have limited robustness and often remain confined to controlled environments or specific applications, resulting in continued reliance on manual counting in most parts of the world.

In recent years, with the advancement of computer vision technology, Convolutional Neural Networks (CNNs) have been demonstrated to be highly effective in addressing a wide range of visual problems, making them a key factor in the success of deep learning (LeCun et al., 2015). With the widespread availability of low-cost digital cameras and high-performance graphics processing units, deep learning methods have enabled the shift from traditional manual labor to automated solutions for plant vision application. Various methods have been developed for different applications, such as estimating ear density (Xiong et al., 2019), locating cotton balls (Sun et al., 2022), detecting maize tassels (Yu et al., 2023), and counting rape flower clusters (Li et al., 2023). Lu et al. (2023) proposed the YOLOv8-UAV model, which introduces a simple and effective up-sampling process. Channel suppression is performed after each up-sampling step to eliminate feature redundancy, enhancing the perception of small-scale objects. Yang et al. (2022b) presented a method for calculating soybean pod length and width based on the Mask R-CNN network structure. This approach enables rapid segmentation and effective calculation of pod shape features from images. Ye et al. (2024) modeled a general network architecture, PlantBiCNet, which employs a bi-directional cascade decoding approach to fully utilize high-level semantic and low-level spatial information, markedly enhancing the detection and counting performance for five different crops. Shortly after, Ye and Yu (2024) considerably improved tassel detection performance in UAV scenes by fusing global and local information in images and modeling an encoder network with 16x downsampling layers, termed FGLNet. Li et al. (2019) proposed a deep learning model called “Two-Column Convolutional Neural Network” for soybean seed counting based on pod images. This model consists of two parallel convolutional neural networks designed for automatic seed counting in soybean pods.

While there have been some successes in the field, research on soybean pod recognition and counting remains relatively limited. To enhance accuracy, Xu et al. (2023) utilized a deformable attention recursive feature pyramid network to improve pod counting precision. Guo et al. (2021) directly detected soybean pods on the entire plant, incorporating the K-means clustering algorithm and an improved attention mechanism module, achieving an average accuracy of 80.55%. Zhao et al. (2022) introduced a method called P2PNet-Soy, which resulted in a significant reduction in the mean absolute error (MAE) in soybean seed counting and localization, decreasing from 105.55 to 12.94. Yang et al. (2022a) employed a large-scale Transformer for pod recognition. On the other hand, McGuire et al. (2021) deployed soybean pod counting on a small mobile robot, walking autonomously in the field, and collecting pod images for analysis. They reported a correlation of 0.88 between automated pod counting and manual counting. Although these existing object detection methods have improved both counting accuracy and speed, their performance still falls short of practical application requirements for pod recognition and counting, especially in the following scenarios:

1) Pod Occlusion: Due to the clustered growth nature of pods, a single region in a soybean image often contains a large number of pods, and these pods frequently overlap with each other. This results in convolutional neural networks inevitably introducing noise during pod feature extraction, affecting the final detection accuracy.
2) Uneven Pod Distribution: The branched structure of soybeans leads to notable differences in pod density across various regions. Models need to adapt to different density distributions in different areas.

Moreover, these methods often require substantial computational resources, rendering them inefficient and inadequate for real-time tasks. In recent research, Xiang et al. (2023) introduced an improved YOLO POD model capable of accurate soybean pod recognition and counting without compromising inference speed. It achieved an impressive fit with an R^2^ of 0.9666. Notably, the YOLO POD model performs approximately 394.9G floating-point operations per second (FLOPs), requiring 0.462 seconds for inference on each high-resolution image. This implies a processing rate of only about two images per second, highlighting the efficiency limitations that restrict its throughput when handling large quantities of high-resolution images. FLOPs are a standard metric used to measure a model’s computational complexity and resource requirements. Higher FLOPs values indicate that the model demands more powerful GPU or CPU support during runtime and inference, which can pose challenges in practical model deployment.

In modern high-throughput plant phenotyping systems, rapid processing of high-resolution images is of paramount importance. While soybean pod recognition and counting have achieved impressive accuracy, improving efficiency to meet the demands of high-resolution image analysis is a natural and critical research focus.

To better address the challenges mentioned above, we propose a deep learning-based soybean pod counting and localization method called PodNet. It leverages a lightweight backbone CSPDarknet (Bochkovskiy et al., 2020), and further enhances feature information decoding using an asymptotic feature pyramid network (AFPN) (Yang et al., 2023), which supports direct interaction between non-adjacent levels. Our evaluation is based on publicly available soybean pod detection and counting datasets (Xiang et al., 2023). Currently, YOLO POD reports state-of-the-art counting performance, but it relies on a substantial number of parameters and floating-point operations. Remarkably, our proposed PodNet achieves nearly the same level of accuracy as the SOTA model YOLO POD while having only 2.48M parameters, which is approximately 1/22 of the YOLO POD model’s parameters. Furthermore, the computational complexity, measured in FLOPs, has been reduced by over 10 times. This means that the PodNet model is relatively more lightweight in terms of size, with a smaller parameter footprint.

**Figure 1:**
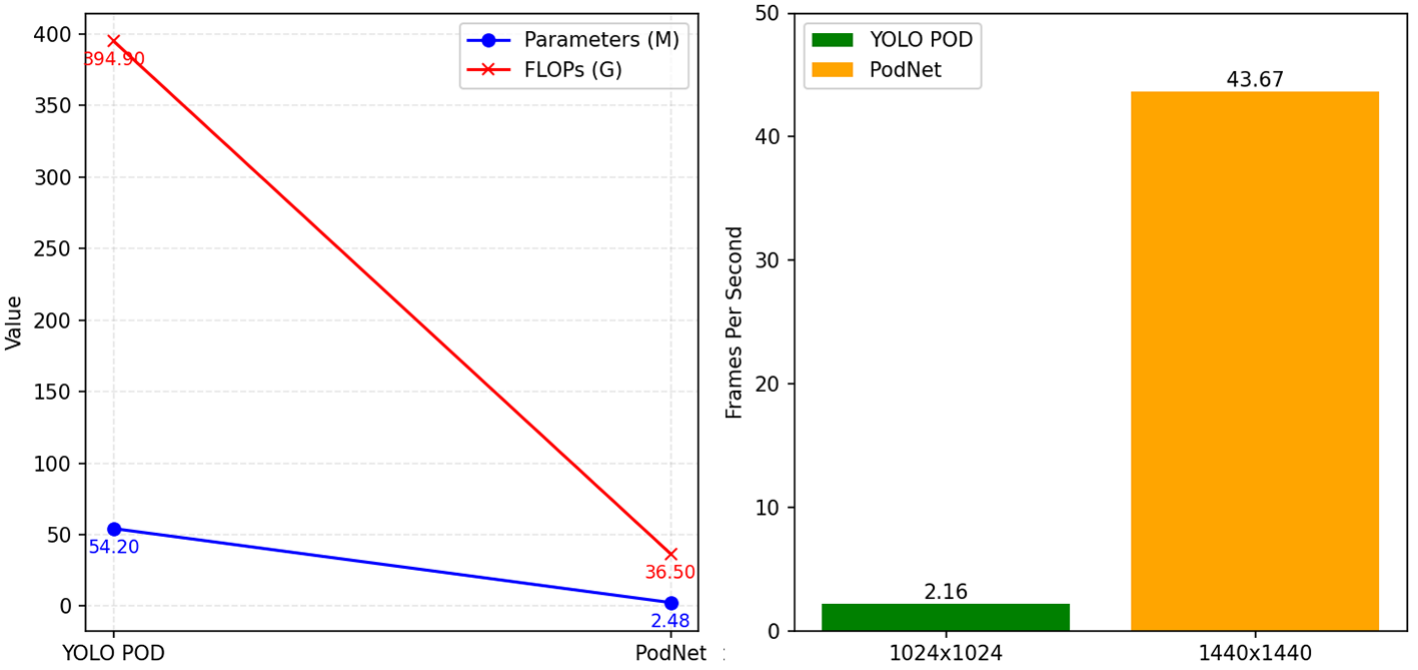
Comparison of FPS, Parameters, and FLOPs between YOLO POD and PodNet.

In the comparison of Frames Per Second (FPS), PodNet performs even more impressively. Even when performing inference at a higher resolution (1440×1440 image resolution) compared to YOLO POD, PodNet still reports a frame rate of 43.67 FPS on a budget-friendly GTX1080Ti GPU. Its efficiency exceeds YOLO POD by an order of magnitude and is sufficient for real-time deployment of models on inexpensive devices. Their parameter comparison is illustrated in **Figure 1**.

Overall, the main contributions of this paper are three-fold:

1) PodNet: A novel deep convolutional network with a lightweight encoder and an effective decoder for decoding encoded features.
2) Efficiency Improvement: PodNet achieves an efficiency improvement of over an order of magnitude when compared to the state-of-the-art models used for soybean pod counting.
3) Real-time Efficiency on Low-cost Devices: It reports high real-time efficiency on inexpensive devices, providing an effective and cost-efficient solution for modern high-throughput plant phenotyping platforms.

The layout of this paper is as follows:

In Section 1 (this section) introduces the research background and highlights the problem statement. Section 2 provides a detailed introduction and description of the proposed PodNet model. In Section 3, experiments are conducted, and comprehensive comparative analyses with other models are performed across various dimensions. Section 4 delves a discussion of the research, and Section 5 concludes the study while outlining future research directions.

## 2 Materials and methods

In this section, we describe the essential data collection and preprocessing steps for this project. What’s more, we provide a detailed introduction to the deep learning framework employed for soybean pod counting and locating, which enables accurate pod counting and localization.

### 2.1 Pod counting datasets

The dataset used in this study is sourced from Xiang et al.’s dataset (Xiang et al., 2023), which was made publicly available in their research and is suited for soybean pod counting tasks. The first dataset is Chongzhou dataset, which is collected in 2021 at Sichuan Agricultural University Chongzhou Experimental Base (103°40’E, 30°39’N). These images were taken by Canon 700D with a resolution of 4752×3168 pixels, a total of 570 images were acquired. The second is the Renshou dataset, collected in 2021 from Renshou Farm of Sichuan Agricultural University (104°08’E, 29°59’N), taken by Canon 750D with a resolution of 5184×2196 pixels, a total of 878 images were acquired. During the capture process, the soybean pods were placed on non-reflective black absorber cloth.

For model training and evaluation, we use the Chongzhou dataset containing 570 images for training and validation. At the same time, the Renshou dataset containing 878 images is used to test the performance of the model.

Due to the availability of bounding box annotations in this dataset, each pod has been labeled with a bounding rectangle. These annotation data serve as the foundation for our model training, so we only performed a verification check on pod annotations using LabelImg (Tzutalin, 2022) to ensure their accuracy. In each image, the number of soybean pods varies from 15 to 185.

**Figure 2:**
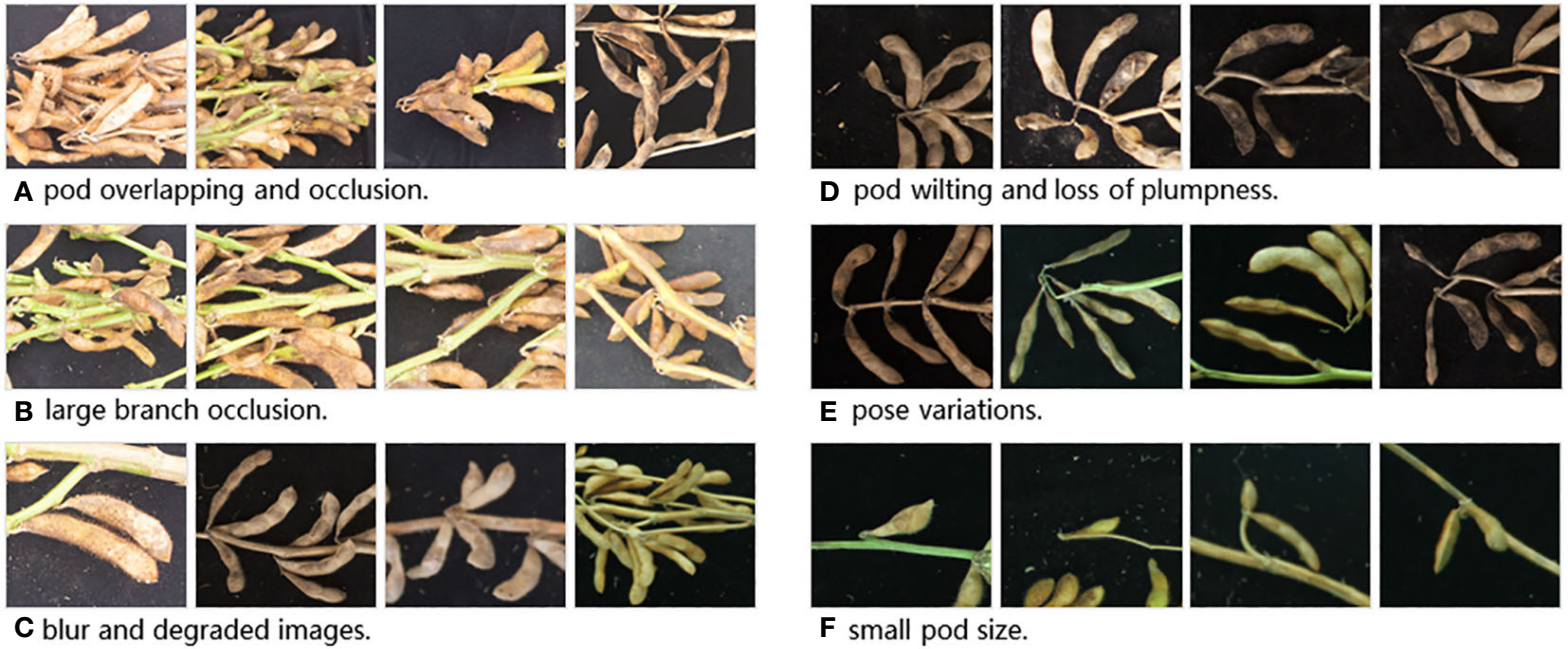
Six Main Challenges in Pod Counting Task **(A–F)**.

We believe that evaluating on these datasets can greatly illuminate the applicability of plant counting tools in real-world scenarios. Plant counting, nonetheless, is a challenging task, especially when dealing with individual plants like soybean pods, which are small and subject to various imaging factors. We summarize the dataset’s characteristics and the key challenges of plant counting into several types, as shown in **Figure 2**.

a) Pod Overlapping and Occlusion: Soybean pods can be obscured by both other pods and plant stems, leading to mutual occlusion between pods and between pods and branches.
b) Large Branch Occlusion: Pods may be hidden behind or obstructed by thicker plant stems, making it challenging for humans to discern the exact number of pods.
c) Blur and Degraded Images: Despite high image resolution, factors such as inaccurate focus or lens surface imperfections like dust can result in image blurring and degradation.
d) Pod Wilting and Loss of Plumpness: Pods may appear wilted and less plump, especially those at the tips of branches, often due to insufficient moisture, causing them to lose their normal color and fullness.
e) Pose variations: Pods may exhibit different poses when captured from various angles, making them not necessarily flat and uniformly oriented.
f) Small pod size: Some pods may be underdeveloped or in the early stages of growth, appearing relatively small in the images. Identifying such small pods accurately requires a model with high resolution and sensitivity.

It is precisely because of these challenges that we must handle these limitations with care to ensure that our model exhibits better robustness and strong generalization performance. In our research, we have successfully overcome these challenges and achieved satisfactory experimental results.

### 2.2 Model architecture

**Figure 3:**
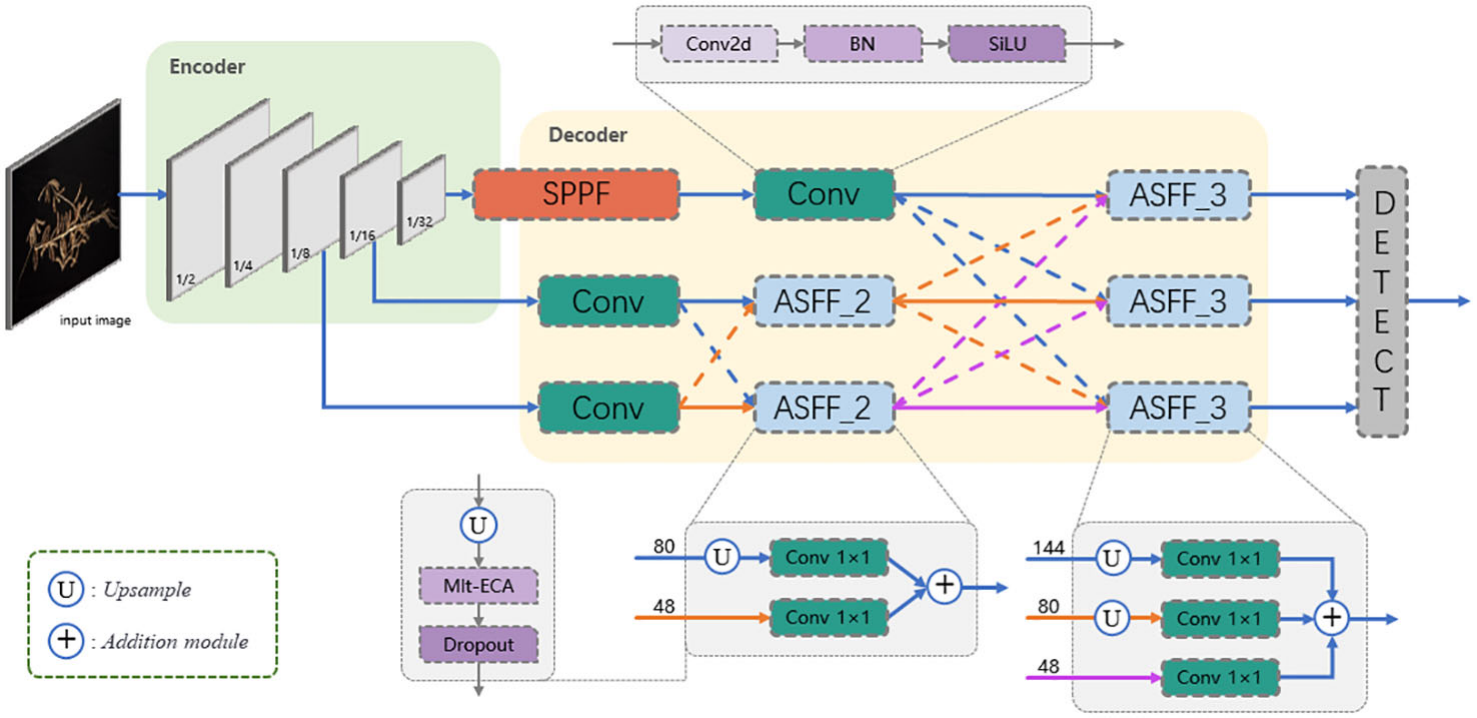
Architecture of PodNet. The number in front of the arrow indicates the number of channels in the input.

Estimating soybean seed yield through automatic counting and locating of pods is a challenging computer vision task. The complexity of this problem comes from various factors such as cluttered visual environments, occlusion of pods in the image, and illumination variations. However, the images in the dataset have already been taken and cannot change their angle, thus we will take a more optimized network model to solve these problems. Considering the deployment requirements of edge devices in the background of plant science, we focus on their lightweight in the model architecture design, and effectively design the detection network structure, making the detection network structure more comprehensive and detailed, especially suitable for identifying small and dense plant objects in the image. As shown in **Figure 3**, in the following we present the global architecture of PodNet and its optimization.

1) Encoder: The role of the encoder is to map the input RGB image into feature maps. We employ CSPDarknet (Bochkovskiy et al., 2020) as the backbone feature extraction part of the encoder. CSPDarknet is a widely validated and used backbone network known for its high efficiency and outstanding accuracy (Lu et al., 2023; Ye et al., 2023; Ye and Yu, 2024; Ye et al., 2024). The entire encoder consists of 5 convolution layers and 4 feature extraction layers, specifically defined as C3(16)-C3(32)-M(32)-C3(64)-M(64)-C3(128)-M(128)-C3(256)-M(256)-S5(256). Here, Ck(m) denotes a 2D convolutional layer with m channels and a k×k filter, all with a stride of 2. M(k) represents a feature extraction layer with k output channels, and the final S5(256) is an Spatial Pyramid Pooling - Fast (SPPF) (Jocher, 2022) module with an input channel count of $C_{input}=256$ and a kernel size of $K_{SPPF}=5\times 5$ .

As described above, given an input image $I\in R^{H\times W\times 3}$ , through the transformation of the encoder, the input image undergoes a 32-fold down-sampling, and the feature maps are reduced to 1/32 of the original image. By inserting the CSPLayer (Wang et al., 2020) at different stages to extract features, each stage respectively outputs feature maps with 32, 64, 128 and 256 channels respectively, and the mapping information is used as the encoding set for the input image. The feature map will be used in Decoder to obtain richer gradient information.

In the final stage of the Encoder, we combine the SPPF module with the CSPDarknet structure to obtain multi-scale feature representations and address the issue of reduced spatial resolution introduced by the SPPF module. Additionally, following the SPPF module, we introduce Convolutional (Conv) layers to extract fine details and enhance feature representation, thereby significantly improving the model’s performance, especially in tasks requiring high spatial resolution. In general, such an Encoder helps improve object detection performance, especially when dealing with challenges like occlusion.

2) Decoder: The role of the decoder is to combine and decode the features from the Encoder, mapping them to the final object detection output. In PodNet, we use the Adaptively Spatial Feature Fusion (ASFF) proposed by Liu et al. (2019) for feature combination. ASFF adaptively fuses spatial features from different stages to gain richer contextual information. ASFF_2 combines features from the first and second convolutional layers, each with 80 and 48 channels, respectively. ASFF_3 further fuses feature from three stages, each with 144, 80 and 48 channels. The selection of these channel numbers aims to balance model complexity and performance, obtaining a more comprehensive extraction of high-level semantic information. Specifically, smaller output channel numbers make the model more lightweight but may lead to performance degradation, while larger output channel numbers provide richer feature representations at the expense of increased computational burden. Including 144 channels allows the model to capture more high-level semantic information, while 80 and 48 channels respectively cater to intermediate and low-level details. They will be applied in the following ASFF module formulas Equations 1, 2:


(1) ASFF2 (Ip1 ,Ip2 )=σ(α2 · Ip1 +(1−α2 )·Ip2 )

(2) ASFF3 (Ip1 ,Ip2 ,Ip3 )=σ(α3 ·Ip1 +β3 ·Ip2 +(1−α3 −β3 )· Ip3 )

Among them, Ipk represents the input feature maps from the k stage, σ is the activation function, sigmoid is used to ensure outputs between 0 and 1, and $\alpha_{2}\;$ , $\alpha_{3}\;$ are adaptive weights that adjust the fusion ratio between two input feature maps.

Taking ASFF_2 as an example. If we designate the post-fusion feature map as L2, we can mark the three spatial weights from the forward propagation of F1, F2, and F3 to L2 are marked as α2 , β2 and γ2 , respectively. This is expressed as the following formula (Equation 3):


(3) $${\text{L}}_{ij}^{2}=\alpha_{ij}^{2}\cdot F_{ij}^{1\to 2}+\beta_{ij}^{2}\cdot F_{ij}^{2\to 2}+\gamma_{ij}^{2}\cdot F_{ij}^{3\to 2}$$

Where $F_{ij}^{1\to 2}$ and $F_{ij}^{3\to 2}$ represent the results of scale transformation, which convert the feature vectors at position (i, j) in F1 and F3 to the same resolution and channels as F2. It is noted that *a^2^_ij_*, $\beta_{ij}^{2}$ and $\gamma_{ij}^{2}$ are scalar variables and are defined as follows by using the softmax function (Equations 4–6):


(4) $$\alpha_{ij}^{2}=\frac{e^{\lambda_{\alpha_{ij}}^{2}}}{e^{\lambda_{\alpha_{ij}}^{2}}+e^{\lambda_{\beta_{ij}}^{2}}+e^{\lambda_{\gamma_{ij}}^{2}}}$$

(5) $$\beta_{ij}^{2}=\frac{e^{\lambda_{\beta_{ij}}^{2}}}{e^{\lambda_{\alpha_{ij}}^{2}}+e^{\lambda_{\beta_{ij}}^{2}}+e^{\lambda_{\gamma_{ij}}^{2}}}$$

(6) $$\gamma_{ij}^{2}=\frac{e^{\lambda_{\gamma_{ij}}^{2}}}{e^{\lambda_{\alpha_{ij}}^{2}}+e^{\lambda_{\beta_{ij}}^{2}}+e^{\lambda_{\gamma_{ij}}^{2}}}$$

In Equations (4–6), we have $\alpha_{ij}^{2}+\beta_{ij}^{2}+\gamma_{ij}^{2}=1$ , and $\alpha_{ij}^{2}$ , $\beta_{ij}^{2}$ and $\gamma_{ij}^{2}$ are all constrained within the range [0, 1]. The parameters $\lambda_{\alpha_{ij}}^{2}$ , $\lambda_{\beta_{ij}}^{2}$ , and $\lambda_{\gamma_{ij}}^{2}$ serve as control factors for these three weights. We compute them using 1×1 convolutions, and they can thus be learned through standard back-propagation.

As shown in **Figure 3**, PodNet combines features from two or three levels of the encoder. ASFF can allocate different spatial weights to features from different levels, enhancing the importance of crucial levels and reducing the influence of conflicting information from different levels. Beyond that, we have incorporated a non-reduction local cross-channel interaction strategy called the Multi-Efficient Channel Attention (Mlt-ECA) module proposed by Yu et al. (2023). It generates feature weights by performing 1D convolutional operations, where the kernel size K is adaptively determined by the channel dimension C and defined as (Equation 7):


(7) $$K={\left|\frac{\log_{2}\left(c\right)+\beta }{\alpha }\right|}_{odd}$$

α and β are adjustable hyperparameters, odd indicates that k is odd. Observing that the soybean pods in the dataset have small areas and a substantial amount of background, and recognizing that the background does not contribute to pod calculation, it makes sense to apply the Mlt-ECA approach. This helps reduce the impact of the background, allowing the model to ignore background features and focus more on the essential pod-related information. This application has indeed improved the model’s performance to some extent.

Next, the decoded feature maps from different stages are merged into the original feature map, through the regression branch, predict the distances between each anchor point and the four edges of the object bounding box, thereby determining the position of the object. The operation of a regression branch is defined as follows (Equations 8–11):


(8) $${\hat{b}}_{x}\;=Sigmoid\left(r_{x}\right)\cdot b_{w}+c_{x}$$

(9) $${\hat{b}}_{y}=Sigmoid\left(r_{y}\right)\cdot b_{h}+c_{y}$$

(10) $${\hat{b}}_{w}=exp\left(r_{w}\right)\cdot b_{w}$$

(11) $${\hat{b}}_{h}=exp\left(r_{h}\;\right)\cdot b_{h}$$

where ${\hat{b}}_{\text{x}}$ , ${\hat{b}}_{\text{y}}$ , ${\hat{b}}_{\text{w}}$ and ${\hat{b}}_{\text{h}}$ represent the predicted values for the center coordinates (x, y), width w and height h of the object bounding box, *r_x_*, $r_{\text{y}}$ , $r_{w}$ and $r_{\text{h}}$ are the outputs of the regression branch, and $b_{\text{w}}\;$ , $b_{\text{h}}\;$ , $c_{\text{x}}\;$ and $c_{\text{y}}\;$ are constants representing the anchor’s width, height and center coordinate offset.

Finally, Non-Maximum Suppression (NMS) is executed to filter the generated prediction boxes to eliminate redundant detection results. NMS is an important step for filtering generated prediction boxes to eliminate redundant detection results. It works by the following formula (Equation 12):


(12) $$NMS\left(boxes,Th\right)\;=\;\{\begin{array}{c} boxes\left[i\right],\;if\;IoU\left(boxes\left[i\right],\;boxes\left[j\right]\right)\leq Th,\;\forall j>i\\ discard,\;otherwise \end{array}$$

Th represents the threshold value, IoU[boxes(i),boxes(j)] denotes the Intersection over Union (IoU) between the i-th and j-th boxes. If the IoU is greater than the threshold value, the i-th box will be retained, otherwise, it will be discarded.

In summary, these simple modifications enhance the model’s perception and expressive capabilities by implementing multi-scale feature fusion, context aggregation, and introducing channel attention. Moreover, the model’s decoder leverages sufficiently deep encoding feature layers from the encoder to respond to more abstract information, combined with an adaptive strategy to restore spatial resolution and fuse feature layers from non-adjacent levels. This enables the model to better adapt to the detection of small and crowded objects.

### 2.3 Loss function

Loss functions, also known as objective functions or cost functions, are methods used to evaluate the difference between model predictions and actual results. The choice of loss function directly impacts the training effectiveness and final performance of the model. We use two simple loss functions to guide the bounding box regression in PodNet.

#### 2.3.1 Classification loss

The classification loss is based on the Binary Cross-Entropy (BCE) function, which is a common binary classification loss used to measure the difference between the predicted probability distribution and the actual labels. It focuses on two classes (typically foreground and background) and is easy to optimize. It is defined as (Equation 13):


(13) $$L_{cls}\left(y,p\right)\;=\;-\left[y\log\left(p\right)+\left(1-y\right)\log\left(1-p\right)\right]$$

Where y is the actual label (0 or 1), p is the predicted probability value (between 0 and 1). The BCE loss penalizes the difference between the predicted probability and the actual label to optimize the model’s classification performance. When the predicted probability is close to the actual label, the loss function has a smaller value, and when the predicted probability deviates from the actual label, the loss function has a larger value. Therefore, the BCE loss can effectively guide the model training to improve classification accuracy.

#### 2.3.2 Localization loss

The localization loss is based on the IoU function supervision. IoU directly measures the overlap between the predicted bounding box and the actual bounding box and is intuitive and interpretable. It is described as (Equation 14):


(14) $$L_{iou}\left(A,B\right)\;=\;\left(A\cap^{\;}B\right)/\left(A\cup^{\;}B\right)$$

Where A represents the area of the predicted bounding box, B represents the area of the actual bounding box, $\cap^{\;}$ represents the intersection of the two regions, and $\cup^{\;}$ represents the union of the two regions. Compared to coordinate-based loss functions, IoU loss is more stable for predicting box position and shape changes. In practical applications, IoU-based loss functions often converge faster and exhibit better robustness.

Finally, combining the two losses gives the total loss of PodNet: $L_{pod}\;=\;L_{cls}+L_{iou}$ . This approach simultaneously considers both classification and localization, allowing the model to strike a balance between classification accuracy and localization precision, ultimately achieving better overall performance.

## 3 Experiments

In this section, we will first introduce the evaluation metrics and experimental details. Then, we will report the performance and compare the proposed PodNet model with existing methods. According to different occlusion degree, we use density map to show the robustness of PodNet in complex cases. Finally, we will conduct ablation experiments to demonstrate the significance of key design choices.

### 3.1 Model training

Our experiments were implemented using the PyTorch deep learning framework (Paszke et al., 2019) and accelerated with CUDA. We used 570 images from the Chongzhou dataset, all of which were used for training and validation. Due to the high resolution of the samples, we resized the input images to 1440×1440 pixels.

During the training process, we employed Stochastic Gradient Descent (SGD) (Loshchilov and Hutter, 2016) as the optimizer, with learning rate, weight decay, and momentum configured as 0.01, 0.0005 and 0.8, respectively. The batch size was set to 8 images per batch. The hardware used for the experiments consisted of an Intel(R) Core (TM) i5-13400F and an NVIDIA GeForce GTX 3090 GPU, with CUDA version 11.8 and CUDNN version 8.9.5 for deep neural network acceleration.

After configuring the relevant parameters, the PodNet model is optimized for 80 epochs on the dataset, taking into consideration the convergence speed. To ensure the robustness of model training, strategies such as color distortion, random scale transformation, and mosaic data augmentation were employed.

### 3.2 Comparison with different object detection methods

To evaluate the superiority of the PodNet model, after completing model training, we compared its detection performance with four state-of-the-art object detection methods. The test images were based on 878 images from the Renshou dataset, each of which was different from those used in the training Chongzhou dataset. To ensure fairness and objectivity of the results, we used the same training and test sets to train and test these models. The methods included YOLOv8 (Jocher et al., 2023), CenterNet (Duan et al., 2019), Faster R-CNN (Ren et al., 2017), TasselLFANet (Yu et al., 2023), FCOS (Tian et al., 2019) and EfficientDet (Tan et al., 2020). Evaluation metrics were primarily based on precision (P), recall (R), mean average precision (mAP@0.5), mAP@0.5:0.95 and F1 (F1-score).

Precision (P) represents the proportion of correctly predicted objects to all predicted objects, while recall (R) represents the proportion of correctly predicted objects to all actual objects. They are defined by the following formulas (Equations 15–18):


(15) $$Precision=\frac{TP}{TP+FP}$$

(16) $$Recall=\frac{TP}{TP+FN}$$

(17) $$mAP=\frac{1}{n}\sum_{1}^{n}P\left(R\right)d\left(R\right)$$

(18) $$F1=2\times \frac{Precision\times Recall}{Precision+Recall}$$

Where TP (True Positives), FP (False Positives), and FN (False Negatives) denote the quantities of true positives, false positives, and false negatives, respectively. “TP + FP” represents the total number of detected soybean pods, while “TP + FN” represents the total number of actual soybean pods in the images. mAP@0.5 indicates the average precision at an IoU threshold of 0.5. mAP@0.5:0.95 represents the average of mAP at different IoU thresholds (from 0.5 to 0.95 in step of 0.05). F1 evaluates the performance of the method by balancing the weight of accuracy and recall rate.

In **Table 1**, we present a comparison of evaluation metrics for different object detection models in the soybean pod counting task.

**Table 1:** Comparison of Evaluation Metrics for Different Models.

Model	P	R	F1	mAP@0.5	mAP@0.5:0.95
YOLOv8	0.842	0.655	0.74	73.4%	39.4%
CenterNet	0.646	0.561	0.60	52.6%	21.5%
Faster R-CNN	0.481	0.447	0.46	36.0%	11.3%
TasselLFANet	0.833	0.679	0.75	71.5%	34.3%
EfficientDet	0.113	0.169	0.14	4.92%	4.6%
FCOS	0.638	0.612	0.62	57.3%	46.4%
PodNet	**0.874**	**0.756**	**0.81**	**82.8%**	**49.9%**

The best performance is in boldface.

Obviously, the PodNet model outperforms other methods to varying degrees. In general, as recall (R) increases, precision (P) tends to decrease, and a balance needs to be struck between them. FCOS model uses fixed anchors to represent the size and shape of objects, employing a direct regression approach for the object’s center point during regression. This approach may not accurately handle variations in rotation angles. The EfficientDet model demonstrates minimal loss during training and validation but performs poorly during testing. EfficientDet achieves efficient object detection by jointly scaling the model’s resolution, depth, and width. Yet this scaling may not be as effective for small and dense soybean pods, as the size and density of these objects may negatively impact the model’s recognition performance. YOLOv8 exhibits excellent real-time performance and simplicity in a single forward pass, introducing detection heads of different scales. However, it offers lower accuracy compared to PodNet, especially with marked room for improvement in recall (R). CenterNet simplifies the object detection task by representing objects as center points, reducing the complexity of bounding box regression. Nevertheless, it may miss small and densely distributed objects due to the interference of dense objects and background information, as well as extensive pod overlaps. Faster R-CNN achieves high-precision object detection performance using a two-stage network with RPN. Whereas it relies solely on the final layer of the convolutional network, which often results in feature maps that are too small. This leads to a rapid decline in recognition performance for small objects, making subsequent detection and regression less effective. For botanical applications, TasselLFANet takes into account plant structural and morphological characteristics. In our experiments, nonetheless, it did not perform very well in cases with dense distribution and frequent occlusion of soybean pods. This may be because it was designed to focus on fitting maize tassels in field scenarios, which are far less dense than soybean pods.

PodNet surpasses other models to varying degrees in multiple metrics, exhibiting higher accuracy in recognizing soybean pods relative to other models. Importantly, it maintains a high level of performance even under the stricter evaluation condition of mAP@0.5:0.95, that’s 10% more than the second-place YOLOv8. This indicates that our model has more accurate localization performance and better robustness.

### 3.3 Comparison with state-of-the-art

After the performance comparison in detection, we further compared it with the current state-of-the-art model YOLO POD, which is designed for soybean pod counting. We employed four metrics to evaluate the consistency between predicted values and ground truth, including Mean Absolute Error (MAE), Root Mean Square Error (RMSE), Mean Absolute Percentage Error (MAPE), and the Coefficient of Determination (R²). Specifically, these four metrics are defined as follows (Equations 19–22):


(19) $$MAE=\frac{1}{n}\sum_{i=1}^{n}\left|\hat{y_{i}}-y_{i}\right|$$

(20) $$RMSE=\sqrt{\frac{1}{n}\sum_{i=1}^{n}{\left(y_{i}-\hat{y_{i}}\right)}^{2}}$$

(21) $$MAPE=\frac{1}{n}\sum_{i=1}^{n}\left|\frac{\hat{y_{i}}-y_{i}}{y_{i}}\right|$$

(22) $$R^{2}=1-\frac{\sum_{i=1}^{n}{\left(y_{i}-\hat{y_{i}}\right)}^{2}}{\sum_{i=1}^{n}{\left(y_{i}-\bar{y}\right)}^{2}}$$

The results of counting performance for different models are presented in **Table 2**. From the test results, it is evident that PodNet model’s counting performance is satisfactory. The MAPE and RMSE metrics of PodNet surpass other models, while in the case of R² and MAE, although YOLO POD slightly leads, the margin is small. Looking at the overall numerical values, the PodNet method exhibits the best comprehensive counting performance, comparable to YOLO POD, indicating our ability to achieve highly accurate object detection.

**Table 2:** Comparison of Counting Performance for Different Models.

Model	MAE	RMSE	MAPE	R^2^
YOLOv8	6.16	8.89	10.05%	0.9035
CenterNet	12.67	17.77	18.75%	0.6926
Faster R-CNN	16.56	19.22	34.95%	0.7211
TasselLFANet	5.92	8.83	9.82%	0.9002
EfficientDet	22.74	38.41	29.17%	0.2777
FCOS	11.88	22.00	16.43%	0.6537
YOLO POD	**4.18**	10.04	6.48%	**0.9666**
PodNet	4.52	**7.65**	**6.24%**	0.9500

The best performance is in boldface.

**Figure 4:**
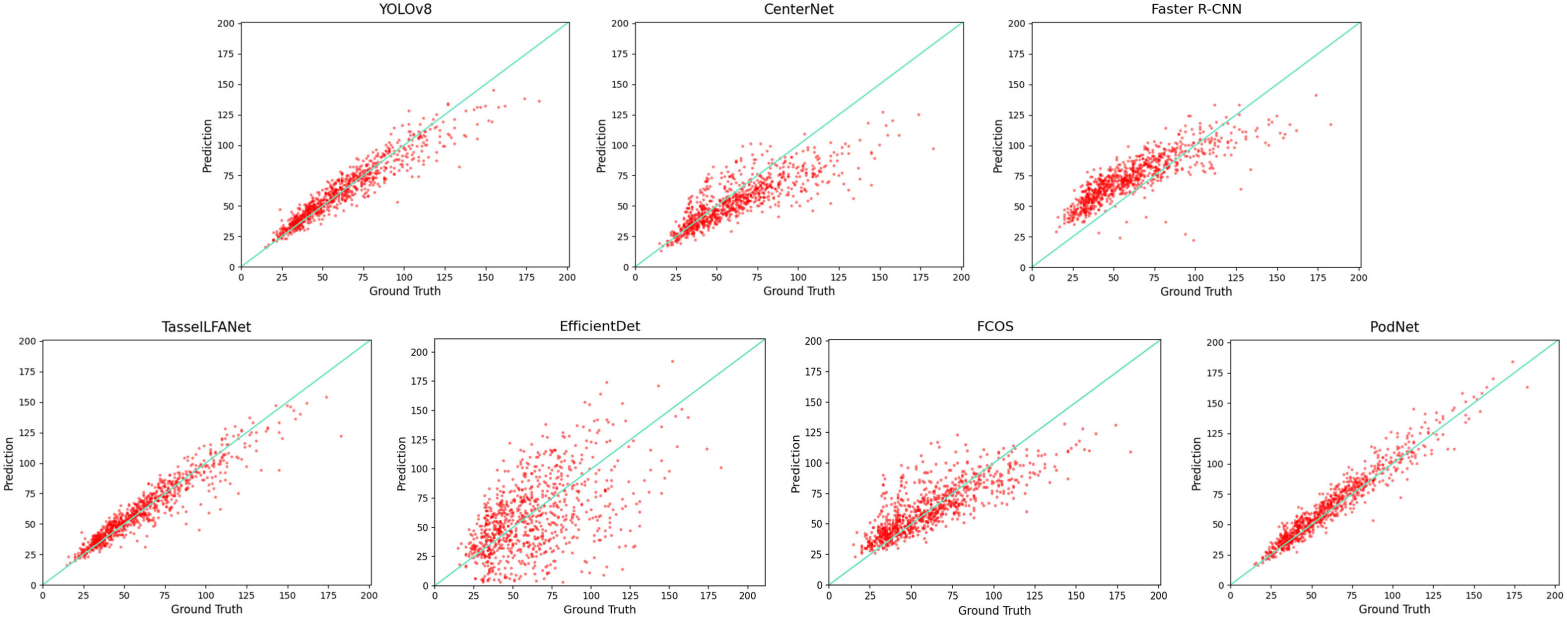
Comparison of Manual Counts and Counts by Different Algorithms.

To visually examine counting errors, we plotted the linear regression relationship between manual counts and algorithmic counts on the dataset, as shown in **Figure 4**. The scatter plot distribution visually demonstrates that our model can capture variations, exhibiting better robustness and generalization. The key lies in the progressive interaction of shallow and deep information, adaptive spatial fusion, improving the model’s performance in both sparse and dense scenes. However, occlusion remains a hot research area in detection methods, and when there are numerous pods in an image, many predicted bounding boxes will be filtered out by the detector’s non-maximum suppression (Neubeck and Van Gool, 2006), leading to underestimation. In relative terms, PodNet can capture additional global information.

In **Figure 4**, we compare manual counts (Ground Truth), counts from various algorithms and predictions made by the models (Prediction). It’s important to note that YOLO POD provided experimental results in the paper, but its engineering was incomplete, making it impossible to reproduce the experiments.

Furthermore, when evaluating the model’s lightweight nature, we compared the parameter count and the floating-point operations per second (FLOPs) between YOLO POD and PodNet, as shown in **Figure 1**. Surprisingly, we found that PodNet has only about 1/22 of the parameter count compared to the YOLO POD model. Moreover, the computational workload (FLOPs) has been reduced by more than ten-fold.

This indicates that PodNet can run in environments with limited hardware resources and has relatively low demands on computational resources. The degree of model lightweighting is particularly important for deploying the model on lightweight platforms, as they are often constrained by computing resources and memory limitations (Ye et al., 2023), such as embedded systems and Jetson Nano development boards. Especially in the field of agriculture, lightweight models can be crucial for certain agricultural managers, effectively reducing their economic burden.

### 3.4 Analysis of visual results

**Figure 5:**
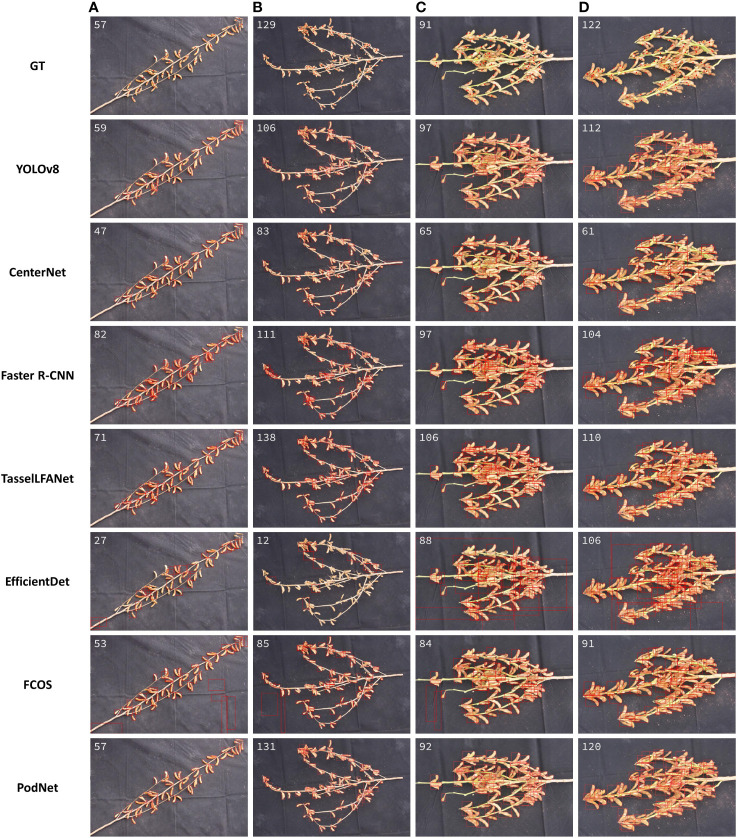
Dataset Visualization Results. **(A, B)** Sparse soybean pods. **(C, D)** Dense soybean pods.

The testing results presented in the previous section represent the overall counting performance of the dataset. To gain a more intuitive understanding of the differences in counting results between different models, we further selected representative soybean plant images, including those with sparse soybean pods (A, B) and dense soybean pods (C, D), as visualized in **Figure 5**. The white number in the top left corner of each image indicates the counting result for that particular image. GT represents ground-truth, which is based on manual annotations from the Renshou dataset, and the red dots denote the center points of each annotated bounding box. The subsequent rows display the counting predictions of each model.

During the experiments, the NMS parameters used for each model were fine-tuned through trial and error. We selected values that struck a balance between preventing the detection of excessive interference and maximizing the identification of correct soybean pods. These parameter settings remained consistent throughout the experiments. For the CenterNet model, in particular, we observed that adjusting the NMS threshold lower resulted in the model detecting many non-soybean pod regions, including branches or even black background areas. This behavior might be related to CenterNet’s design, as it relies solely on the detection of two corner points for object localization, lacking strong overall object information integration capabilities. FCOS model’s use of fixed anchors may not handle well the significant variations in shape, size, and rotation angles of soybean pods, leading to deviations in the regression of the center point. As for the EfficientDet model, it might be due to its complexity or excessive depth, causing the model to overlook smaller objects like soybean pods.

Analyzing the images of sparse soybean pods in **Figures 5A, B** and the images of dense soybean pods in **Figures 5C, D**, we observed an interesting phenomenon: when humans consider the soybean pods sparser, it does not necessarily follow that deep learning models will have better detection results for sparse objects. In the case of dense soybean pods, the inference values may be closer to the ground truth. This observation can be attributed to the following reasons:

1) Contextual Information: Soybean pods are likely to be densely distributed within a certain area. This implies that the model can utilize information from neighboring objects, such as their positions and shapes, to enhance accurate localization and counting. By learning from this contextual information, model improves detection accuracy and reduces the chances of missing or falsely detecting pods.
2) Feature Sharing: In dense object scenarios, objects share more feature information among them, making it easier for the model to learn useful feature representations. This can enhance the model’s generalization performance, enabling it to adapt better to varying object density.

In soybean pod detection, many predicted bounding boxes are filtered out by the detector’s non-maximum suppression and are underestimated. The visual results further confirm that the PodNet model exhibits strong resilience when confronted with variations in soybean pod size and density. In comparison, PodNet can capture additional global information, leading to outstanding performance.

### 3.5 Superiority comparison of pod detection in complex backgrounds

In complex natural environments, the accuracy of a model’s detection can be influenced. Especially when objects overlap with each other, or when objects are covered by other interferences such as branches, the contour of individual pods may be incomplete due to occlusion, making it challenging for the model to detect them. To evaluate the detection performance of the model proposed in this study in complex backgrounds, we selected two sub datasets from the Renshou dataset, one containing images with slightly occlusion (Dataset A, including 15 images), and another containing images with heavily occlusion (Dataset B, also including 15 images).

**Figure 6:**
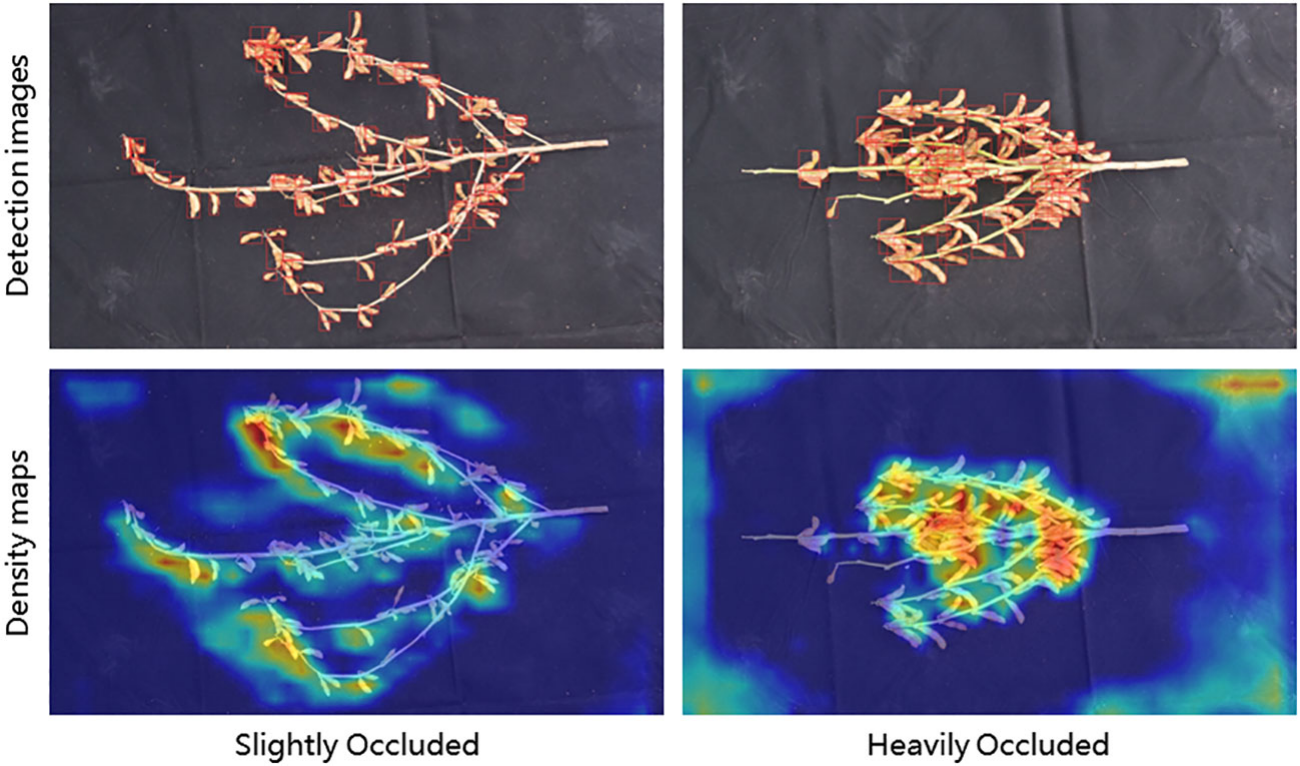
Comparison of Pod Detection Results in Different Occlusion Levels.

We defined the occlusion level as follows: when more than 50% of a pod’s pixel area in an image is covered, it is considered heavily occluded; otherwise, it is considered slightly occluded. After categorizing the images, we used the PodNet model to detect pods in datasets A, B, and the combined A+B. The detection results are shown in **Table 3** and **Figure 6**.

**Table 3:** Comparison of Pod Detection Results in Different Occlusion Levels (Test Set: A = 15 Images with Slightly Occlusion, B = 15 Images with Heavily Occlusion).

Test Set	P	R	F1	mAP@0.5
A	**0.898**	**0.815**	**0.855**	**88.0%**
B	0.836	0.701	0.753	78.1%
A + B	0.868	0.742	0.800	81.7%

The best performance is in boldface.

For slightly occluded pods (Dataset A), the PodNet model achieves an F1-score of 0.855 and an mAP@0.5 of 88.0%. In heavily occluded environments with dense objects (Dataset B), the PodNet model achieves an F1-score of 0.753 and an mAP@0.5 of 78.1%. When the two datasets are combined into one (A+B), the model’s F1-score and mAP@0.5 reach 0.800 and 81.7%, respectively. The results indicate that the PodNet model can effectively detect pods in complex environments.

As shown in **Figure 6**, the colors in the image reflect the density values, with darker colors indicating higher density. In real-world situations, high pod density and issues with photography angles can lead to occlusion, requiring multiple pod counts even for trained professionals to obtain reliable measurements. The experiments demonstrate that the PodNet model can effectively detect and count pods in both slightly and heavily occluded scenarios, meeting the needs of routine agricultural management.

### 3.6 Ablation study

Here, we conducted ablation experiments to validate the effectiveness of incorporating the Mlt-ECA attention strategy into PodNet. We trained the model with the same training parameters and dataset and tested it on the Ren Shou dataset. The experimental results will be presented in **Table 4**. It can be observed that using the Mlt-ECA attention strategy has a positive impact on the model’s performance. This is mainly due to the ability of Mlt-ECA attention to effectively capture critical information from the input data and assign greater weight to this information. It can be observed that using the Mlt-ECA attention strategy has a positive impact on the model’s performance.

**Table 4:** PodNet Conducts Ablation Studies Using the Mlt-ECA Attention Strategy.

At	mAP@0.5	mAP@0.5:0.95	MAE	RMSE	MAPE	R^2^
—	80.8%	47.7%	5.78	7.68	10.24%	0.9354
*✔*	**81.6%**	**47.9%**	**4.70**	**6.50**	**7.99%**	**0.9485**

“✔” means joining the corresponding module and the best performance is in boldface.

## 4 Discussion

This study introduces an advanced soybean pod detection model, PodNet, and demonstrates its performance in complex agricultural environments. Nevertheless, through a detailed analysis of the model’s performance, we identified some challenges in plant counting, particularly in overestimation and underestimation, as illustrated in **Figure 4**. In addition, we observed limitations in the dataset. While PodNet performs well in most cases, it may be impacted under extreme occlusion conditions, necessitating further research and improvement. Specifically, data captured on non-reflective black absorbent cloth may affect the model’s generalization ability. Despite using the Chongzhou and Renshou datasets for training and evaluation, they are relatively small in scale, and larger, more diverse datasets could enhance the model’s comprehensive learning and generalization. Also, in practical applications, the model may face domain transfer issues, where challenges from the dataset may extend beyond the six types mentioned in Section 2.1, leading to a decrease in counting performance in unseen environments or challenges. To address these issues, transfer learning and domain adaptation techniques may be potential solutions (Lu et al., 2017a; 2017b; 2018).

In summary, despite PodNet’s impressive performance in soybean pod detection, we should be cautious in using plant counting tools in practical applications and recommend choosing the right solution for your specific application. Future research directions should focus on addressing the model’s performance in extreme conditions, exploring more sophisticated occlusion handling strategies, validating generalization capabilities for different crops and environments, and emphasizing comprehensive performance comparisons and explanations for hyperparameter selection. This will contribute to enhancing the model’s practicality and robustness, promoting its successful application in a broader range of scenarios.

## 5 Conclusion

Counting soybean pods efficiently and accurately has always been a challenging task, especially for the dense object counting of small, unevenly distributed soybean pods. In this study, we propose a novel deep learning neural network that employs a lightweight encoder and an effective decoder for decoding both shallow and deep information, alleviating the indirect interactions caused by information loss and degradation at non-adjacent levels. Our goal is to address the soybean pod counting and localization problem. Importantly, we consider efficiency improvements, finding a relatively lightweight model with smaller parameter size in terms of dimensions. Compared to YOLO POD, the PodNet model shows significant improvements in both performance and parameter optimization. This improvement is not achieved at the cost of efficiency, as PodNet operates at an order of magnitude higher computational speed than YOLO POD.

Our research is conducted on datasets with various challenges, and we validate its superior performance on multiple performance metrics. Furthermore, we thoroughly validate the effectiveness of dropout strategies and the introduction of the Mlt-ECA attention strategy as optimization techniques for the model. This provides valuable insights for optimizing models to address plant counting and localization problems. We hope this work will further inspire researchers’ interest. Future research could build upon this study by further investigating with more extensive datasets. What’s more, introducing adversarial elements and enhancing and optimizing object detection models could increase their adaptability to different scenarios and changes. This improvement aims to enhance the robustness of the models when facing complex situations like noise and occlusion.

## Data availability statement

The original contributions presented in the study are included in the article/supplementary material. Further inquiries can be directed to the corresponding authors.

## Author contributions

ZHY: Methodology, Software, Funding acquisition, Writing – original draft. YW: Conceptualization, Writing – original draft. JY: Formal analysis, Validation, Writing – review & editing. SL: Investigation, Data curation, Writing – review & editing. DL: Funding acquisition, Project administration, Writing – review & editing. XZ: Validation, Resources, Writing – review & editing. ZMY: Supervision, Project administration, Writing – review & editing. QT: Funding acquisition, Writing – review & editing.
